# The Coronary Dilation Effect of Shen Fu Injection Was Mediated through NO

**DOI:** 10.1371/journal.pone.0092415

**Published:** 2014-03-24

**Authors:** Yu Hong Li, Bin Yu, Zhen Zhen Duan, Olunga Mary Akinyi, Jia Hui Yu, Kun Zhou, Yue Zhang, Xiu Mei Gao

**Affiliations:** 1 State Key Laboratory of Modern Chinese Medicine, Tianjin University of Traditional Chinese Medicine, Tianjin, P. R. China; 2 Ministry of Education Key Laboratory of Pharmacology of Traditional Chinese Medical Formulae, Tianjin University of Traditional Chinese Medicine, Tianjin, P. R. China; 3 Institute of Traditional Chinese Medicine Research, Tianjin University of Traditional Chinese Medicine, Tianjin, China; 4 State Key Laboratory of Modern Chinese Medicine, Ministry of Education Key Laboratory of Pharmacology of Traditional Chinese Medical Formulae, Tianjin University of Traditional Chinese Medicine, Nankai District, Tianjin, P. R. China; VCU, United States of America

## Abstract

**Objectives:**

Shen Fu Injection (SF), which consisted of Red ginseng extraction injection (RG) and prepared aconite extraction injection (RA), is a traditional Chinese medicine mainly used for various cardiac diseases. This study is to analyse SF's effects on cardiac performance and coronary circulation. And the coronary dilating effect and mechanism of the above three injections were also explored.

**Methods:**

Mature male guinea pigs were used as our animal model. We employed two types of perfusion methods (constant pressure and constant flow) *in vitro*, using Langendorff heart preparations to observe the cardiac function and coronary response to SF (1/200). The coronary dilation effects of the above three injections (1/800, 1/400 and 1/200) were recorded at basal coronary resting tone and when coronary vessels were pre-contracted with a thromboxane A2 analogue (U46619), in the presence or the absence of the inhibitor of nitric oxide synthesis (L-NAME, 10^−4^ M), the blocker of Ca^2+^-activated potassium channel(TEA, 10^−3^ M), or the blocker of adenosine triphosphate (ATP)-sensitive potassium channel (glybenclamide) (10^−5^ M).

**Results:**

When perfused with constant pressure, SF significantly increased coronary flow, left ventricular developed pressure (LVDP) and the rate-pressure product (RPP). When perfused with constant flow, SF produced a significant reduction in the coronary perfusion pressure (CPP), LVDP and RPP. The coronary vasodilatation response of the above three injections can be reduced by L-NAME but was unaffected by TEA or glybenclamide when coronary vessels were pre-contracted with U46619 but not at resting tone. SF, RG and RA can all up-regulate eNOS expression in the human umbilical vein cells (EA.hy926).

**Conclusion:**

We demonstrated that SF does not contribute to the inotropic change of myocardium whose improvement is due to alternation of coronary flow. The coronary dilation effect of SF was mediated through RG and RA, via promoting NO release.

## Introduction

Shenfu Injection (SF) has been used in clinic as a medicine to treat cardiac disease including coronary heart disease, myocardial ischemia/reperfusion injury and especially these disease with heart insufficiency or congestive heart failure in China for a long time [1]–[3]. SF was consisted of Red Ginseng extraction and prepared aconite extraction [4]. Red Ginseng (Radix Ginseng Rubra) is the steamed root of Panax Ginseng C. A. Meyer, a perennial herb of the family Araliaceae that has been traditionally used (over 2,000 years) as a medicinal preparation in Republic of Korea, China, and Japan. The basis of the medicinal prowess remains unknown. Its roots and extracts have been used to increase physical strength, prevent aging, and increase vigor [5]. Red ginseng extracts were reported to inhibit intracellular ROS in cultured neurons [6]. Red ginseng marc oil (RMO) inhibited the production of oxidative stress molecules such as nitric oxide through down regulating the level of inducible nitric oxide synthase (iNOs) *in vitro* [7]. Red ginseng can elevate blood pressure through activating vasoconstrictors such as endothelin-1 and angiotensin II [8]. Red ginseng significantly attenuated myocardial ischemic injury by improving cardiac systole function, partly by reducing cardiac Troponin I secretion [9].

The major active ingredients of P. ginseng are the triterpene glycosides also known as ginsenosides, which have a dammarane skeleton [10]. Ginsenosides were a group of compound belongs to Dammarane type which includes Ginsenosides Rb1, Rb2, Rc, Rd, Rg1, Re, Rf, Rg2 and Rg3. Modern pharmacological research shows that ginsenosides can improve ischemic myocardium metabolism, scavenge free radicals, protect myocardial ultrastructure, and reduce Ca^2+^ overload [11], [12]. Chen reported that ginsenoside's cardiac protection effect was partially via promoting releasing Nitric Oxide (NO) from endothelium [13]. In parallel, Deng et al demonstrated that Ginsenoside Rg induced protection against LV hypertrophy is mediated, at least in part, via upregulation of edothelium NO synthase (eNOS) and subsequent endogenous NO production and release [14]. Ginsenoside Re has negative effect on cardiac contractility and autorhythmicity through alternations in cardiac electrophysiological properties [15].

Aconite (Radix Aconiti Lateralis Preparata) who has protective effect on myocardial cells, and it also affects heart rate, rhythm, blood pressure, and hemodynamics [16]. Higenamine (HG) is a potent cardioactive benzylisoquinoline alkaloid isolated from Aconiti tuber. Higenamine can enhance heart contractility, improve coronary circulation, and decrease the effect of acute myocardial ischemia. Higenamine was well known for its inotropic and chronotropic effect in cardiovascular system. It also relaxes isolated rat aorta [17]–[18].

Shen Fu was known to be able to dilate coronary artery by which it might exert its heart protection effect [45]–[46]. Here we reported that administration of Shenfu Injection, Red ginseng extraction injection (RG) and prepared aconite extraction injection (RA) all show coronary artery dilation effect, at least partially through NO release as they all up-regulate eNOS level in endothelium cellsWe also observed increases of the hemodynamics (i.e. LVDP and RPP) induced by SF was the secondary responses to coronary vasodilation.

## Materials and Methods

### 2.1 Animal

Mature male guinea pigs weighing 300–350 g were provided by Beijing Vital River Lab Animal Technology Co. Ltd. Guinea pigs were allowed to eat a standard diet and drink ad libitum, and adapted to the experimental conditions at 22±2°C, humidity 60±5%. This study was carried out in strict accordance with the recommendations in the Guidance Suggestions for the Care and Use of Laboratory Animals issued by the Ministry of Science and Technology of China. The protocols include anesthesia, operation, administration of medication, organ harvest. Body disposal were approved by the Laboratory Animal Ethics Committee of Tianjin University of Traditional Chinese Medicine (Permit Number: TCM-LAEC2013005).

### 2.2 Isolated Langendorff Heart Preparations

Isolated heart experiments were performed in accordance with the methods previously described [19]. Guinea pigs were anesthetized with intraperitoneal injection of sodium pentobarbital (50 mg/kg, i.p.). A thoracotomy was then performed and hearts were rapidly excised into an ice-cold heparinized (5 U/mL) modified buffered Krebs-Henseleit solution (KHs). After removal of lung and surrounding tissue, aortas were immediately cannulated with a needle, which was connected to the Langendorff apparatus to start retrograde perfusion with KHs. Perfusion fluid and bath temperature were maintained at 37°C by a thermostatically controlled water circulator. Hearts were immersed in a water-jacketed perfusate bath maintained at 37°C. The KHs contains (in mmol/L) 118 NaCl, 4.7 KCl, 25 NaHCO_3_, 1.2 MgSO_4_, 1.8 CaCl_2_, 1.2 KH_2_PO_4_, and 11 glucose equilibrated with continuous gassing 95% O_2_ and 5% CO_2_ at 37°C, to yield a physiological pH of 7.3–7.4 [20].

Coronary perfusion pressure (CPP) was measured through a lateral connection in the perfusion cannula connected to a pressure transducer (MLP844 Physiological Pressure Transducer, ADInstruments). The cardiac functions were determined by a modified isovolumetric Langendorff technique [21]. The left ventricular pressure was measured with a water-filled balloon constructed of plastic film inserted into the left ventricle via the left atrium and connected to a pressure transducer. Balloon volume was modified through a stopcock attached to the ventricular pressure transducer using a syringe to maintain a left ventricular diastolic pressure of 5–10 mmHg. Cardiac functions were evaluated upon left ventricular developed pressure (LVDP, left ventricle end systolic pressure minus left ventricle end diastolic pressure), maximal and minimum rate of pressure development (±d*P*/d*t*_max_), and the rate-pressure product (RPP, indicative of cardiac work) calculated as the product of LVDP and HR [22]–[23]. LVDP, ± d*P*/d*t*_max_ and heart rate (HR) were calculated from the left ventricular pressure curve. These parameters were recorded continuously on a computer using PowerLab/8SP Chart 5 software (AD Instruments, Australia).

In constant pressure perfusion mode, CPP was maintained constantly at 70∼80 mmHg. Measuring the Coronary Flow (CF) was allowed by collecting the effluent dripping from the heart [21]. Thus an increase or decrease of CF represents dilatation or constriction of the coronary artery respectively. On the other hand, in constant flow perfusion mode, CF was maintained constantly at 11∼15 mL/min which allow CPP maintained at approximately 70 mmHg. This allowed to measure the CPP (indicative of coronary resistance). An increase or decrease of the CPP indicates constriction or dilatation of the coronary, respectively [24].

### 2.3 Experimental protocol

Cardiac performance: The effects of Shenfu injection (SF) on the cardiac performance were studied in isolated perfused guinea pig model with constant pressure or constant flow. After a 15-minute or more time equilibration, the hearts were perfused with drugs dispersed in KHs at the required concentrations for 10 min. HR, LVDP, RPP, ±d*P*/d*t*_max_ and CF (with constant pressure) or CPP (with constant flow) were measured before and after the application of Shenfu injection.

Vasodilation experiments: After a 15-minute or more time equilibration with constant flow mode perfusion, coronary vasculature was either maintained at resting tone with baseline perfusion pressure of 70∼80 mmHg or precontracted by addition of the thromboxane A2 analogue U46619 (1×10^−8^∼3×10^−8^ M) to achieve a perfusion pressure of approximately 120 mmHg. When the contraction in response to U46619 reached a stable plateau, SF or RG or RA were injected into the perfusion cannula with an infusion pump at a constant rate for 2 minutes to reach final concentrations of 1/200, 1/400 and 1/800 from the commercial injections.

To investigate the underlying mechanisms of coronary effects of these three injections, the responses were recorded in separate experiments in the absence(control) or the presence of the nitric oxide synthesis blocker, *N*-omega-mitro-L-arginine methy1 ester(L-NAME, 10^−4^ M), the Ca^2+^-activated potassium channel blocker, tetraethylammonium (TEA, 10^−3^ M), or the adenosine triphosphate (ATP)-sensitive potassium channel blocker, glybenclamide (10^−5^ M). In each case, antagonists were infused for 5 minutes before pre-contraction of the coronary vasculature by U46619 infusion, and the relaxation responses to these three injections were then recorded as described above. The antagonists perfusions were maintained throughout the experiment. The perfusion pressure was recorded continuously before and after SF, RG, or RA.

Cell cultures: The human umbilical vein cells (EA.hy926) were cultured in DMEM with 10% fetal bovine serum, 100 units/mL penicillin, and 100 μg/mL streptomycin under standard culture conditions (37°C, 95% humidified air and 5% CO_2_). Then the cells were divided into four groups: control group (no administration of medication), SF, RG and RA group (24 hours administration of 1/200 SF, RG or RA injection respectively). Rat Cardiac Microvascular Endothelium Cells (RCMEC) were isolated and cultured as reported [48].

Western blotting: After stimulation of SF, RG or RA of EA.hy926 cells, cells were washed, scraped from dishes, and lysed in RIPA buffer containing a mixture of protease inhibitors (1 mmol/L aprotinin, 20 mmol/L phenylmethysulfonyl fluoride and 200 mmol/L sodium orthovanadate). Proteins were then separated by electrophoresis on SDS-polyacrylamide gel. After the proteins had been transferred onto a PVDF membrane, the blot was incubated with blocking buffer for 1 hr at room temperature and then probed with primary antibody against eNOS overnight at 4°C, followed by incubation with horseradish peroxidase-conjugated secondary antibody for 1 hour.

### 2.4 Drugs and reagents

Shenfu injection, Red ginseng extraction injection and Prepared aconite extraction injection were donated by China Resources Sanjiu Medical & Pharmaceutical Co., Ltd. The main components of shenfu injection include ginsenosides (>0.8 mg/ml) and aconitine (<0.1 mg/ml). The main components of Red ginseng extraction injection include ginsenosides (>0.8 mg/ml). The main components of Prepared aconite extraction injection include aconitine (<0.1 mg/ml). 9, 11-dideoxy-1a, 9a-epoxymethanoprostaglandin F2a (U46619), N-omega-nitro-L-arginine methyl ester hydrochloride (L-NAME),tetraethylammonium chloride (TEA), and 5-chloro-N-[4-(cyclohexylureidosulfonyl) phenethyl]-2-methoxybenzamine (glybenclamide, glyburide) were obtained from Sigma. EA.hy926 cells were commercially available from ATCC. Fetal bovine serum (FBS) and Dulbecco's modified Eagle medium (DMEM) were purchased from Gibco. Antibody against eNOS was bought from Cell Signaling Technology, China (#9586). RIPA buffer was bought from Sigma-Aldrich

### 2.5 Data and statistical analysis

Data are presented as mean ±SD. (Western of EA.hy926 cells data are presented as mean ± SEM). Drug-induecd changes in CF, CPP, LVDP and RPP were compared with a matched control measurement obtained immediately prior to drug perfusion using the paired Student t-test. The coronary dilation effect to SF, RG, or RA at basal coronary resting tone and coronary vessel precontracted with U46619 or treated with antagonists is expressed as the change in CPP. The responses between three injections and between in the absence and presence antagonists were compared using one-way analysis of variance followed by the Dunnett test. Statistic significance was accounted when P<0.05.

## Results

### 3.1 The effects of Shenfu injection on the cardiac performance with constant pressure mode or with constant flow mode in isolated perfused guinea pig hearts

**Figure 1:**
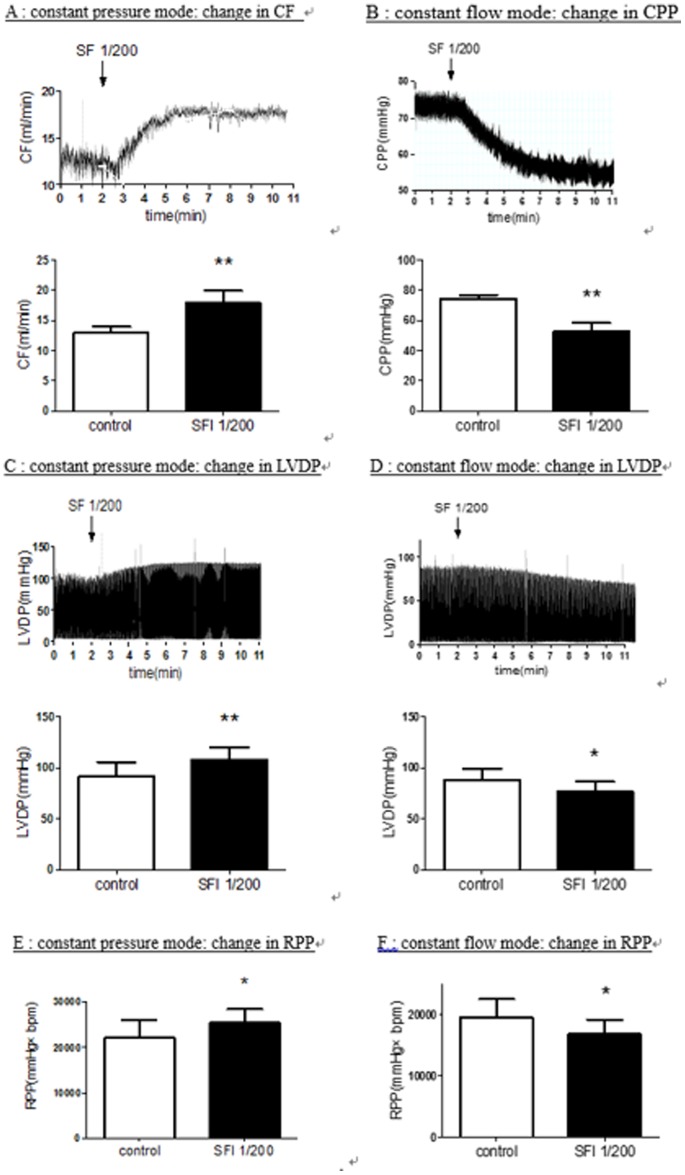
Comparing the Effects of SF injection (1/200 dilution from commercial injections) on the cardiac performance perfused with constant pressure and with constant flow in an isolated perfused guinea pig heart model. KHs with no administration of medication was used as control. A: CF elevated after SF injection was perfused in constant pressure mode. B: CPP dropped after SF injection was perfused in constant flow mode. LVDP (1C) and RPP (1E) increased after SF injection was perfused in constant pressure mode while LVDP (1D) and RPP (1F) decreased after SF injection was perfused in constant flow mode. CF: Coronary Flow, CPP: Coronary Perfusion Pressure, LVDP: Left Ventricular Developed Pressure, RPP: the Rate-pressure Product. Values represent the mean ± SD. *P<0.05, ** *P*<0.01 versus control. (N = 6 in Figure 1A, 1C and 1E, N = 7 in Figure 1B, 1D and 1F).

When the hearts were perfused with constant pressure mode, administration of Shenfu injection (final concentrations 1/200 doses, i.e. 1 ml injection dissolved in 199 ml K-Hs) significantly increased coronary flow from baseline levels (12.93±1.02 ml/min) to (17.83±2.10 ml/min) (Figure 1A). In addition to the primary effects of Shenfu injection on total coronary flow, we also observed modest increases in LVDP and RPP following administration of Shenfu injection. It elevated LVDP (91.38±14.36 vs 108.25±11.66 mmHg) (Figures 1C) and RPP (22045.8±4013.1 vs 25380.6±3005.1 mmHg×bpm) (Figures 1E) without modifying the heart rate (241±19 vs 235±18 bpm). Experimentally, the larger changes in LVDP and RPP correlated closely with the larger increases in coronary flow produced by SF. The changes in LVDP were also accompanied by increases and decreases in +dp/dt_max_ and -dp/dt_max_ (the data not shown). Kinetic analysis of the flow and pressure recordings revealed that the drug-associated changes in coronary flow typically preceded changes in LVDP by 2–3 s. These observations indicates that coronary vasodilation occurred as the primary response to drug administration, followed by secondary responses in LVDP and RPP.

To further elucidate the effects of vasodilation of SF. We also observed the changes of hemodynamics induced by SF in isolated guinea pig hearts perfused with constant flow mode. Administration of SF (final concentrations 1/200 doses) produced a significant reduction in the CPP from the baseline level (74.49±2.52 mmHg) to a lower level (52.68±5.53 mmHg) indicating significant coronary dilatation (Figures 1B). The LVDP and RPP decreased resulted from significant reduced CPP (Figures 1D, 1F). SF caused a distinct vasodilatation in isolated guinea pig hearts perfused with constant pressure mode or with constant flow mode.

### 3.2 The coronary dilation effect of SF, RG or RA in isolated guinea pig hearts perfused with constant flow mode

Shenfu injection is a compound constituted by Red Ginseng extraction injection and prepared aconite extraction injection. We investigate the vasodilator effects of SF/RG/RA respectively in isolated guinea pig hearts perfused with constant flow mode.

**Figure 2:**
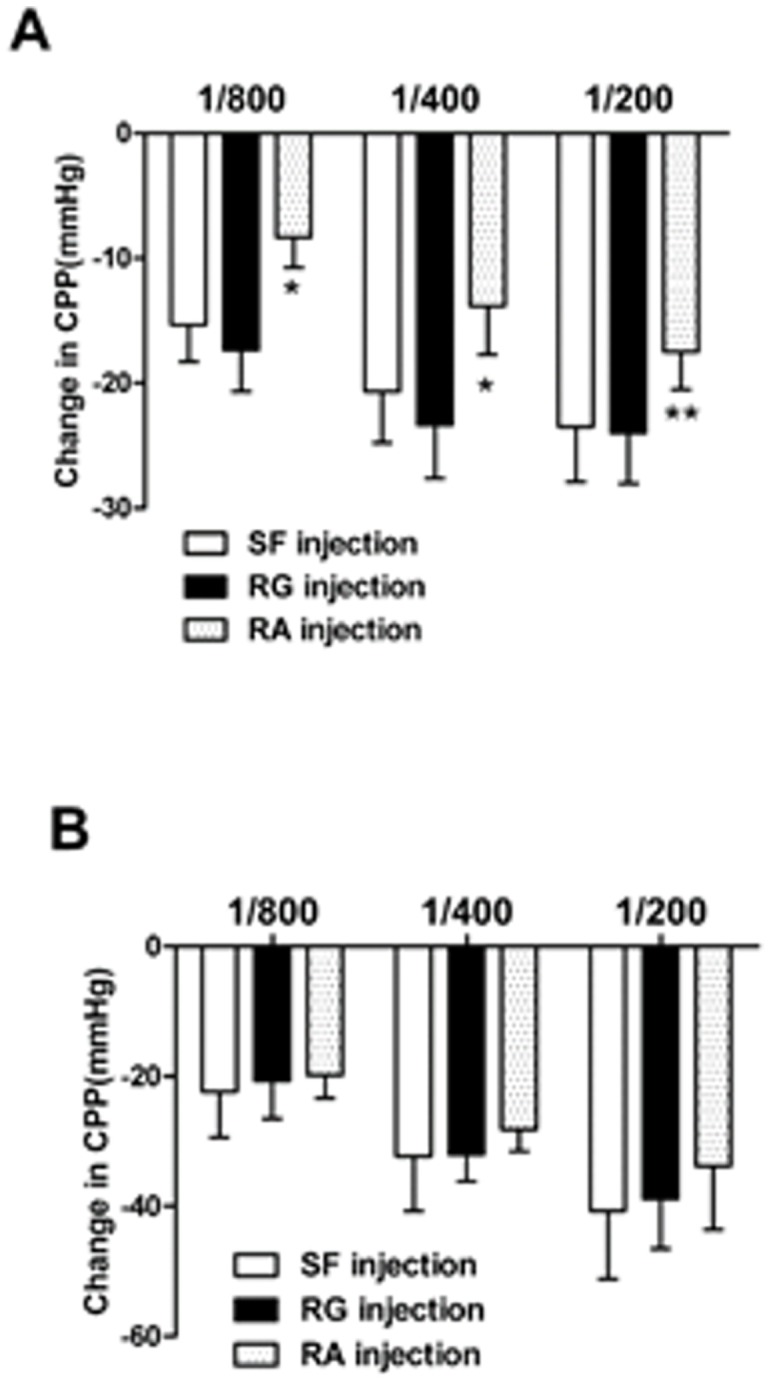
In constant flow mode, comparison of CPP change upon perfusion of SF, RG or RA injection. Three titrations had been tested in each injection (1/800, 1/400, 1/200 dilution from commercial available injections, n = 6). A: RA injection did not bring down CPP as strongly as SF did at basal coronary resting tone. B: Pre-contracted with U46619, the effect of SF, RG and RA show no significant difference in CPP adjustment. Values represent the mean ± SD. *P<0.05, ** *P*<0.01 versus SF injection.

SF/RG/RA injection (n = 6) (final concentratin 1/800, 1/400, and 1/200) caused a concentration-dependent reduction in the coronary perfusion pressure at the basal coronary resting tone. RA injection did not bring down CPP as strongly as SF did at basal coronary resting tone (figure 2A). SF/RG/RA injection had no significant effect on heart rate. The effects on LVDP, +dp/dt_max_ and −dp/dt_max_ produced by RG and RA injection were similar to be produced by SF injection in hearts perfused with constant flow at basal coronary resting tone (table 1).

**Table 1:** Reductions(negative values) or increases (positive values) in HR, LVDP and ±dp/dt produced by administration of SF, RG and RA injection in guinea pig perfused hearts at resting tone(n = 6) and precontracted with U46619(n = 6) with constant flow perfusion.

		1/800	1/400	1/200
Resting tone				
HR (beats/min)	SF	−5.9±5.3	−6.1±5.5	−5.3±13.5
	RG	−2.4±3.5	−0.8±6.7	−6.4±8.5
	RA	−3.7±1.4	−4.7±2.0	−3.3±3.8
LVDP (mmHg)	SF	−1.4±4.1	−3.8±3.9	−7.0±7.5
	RG	1.4±9.9	−8.7±8.4	−10.0±4.9
	RA	−0.1±2.5	−2.2±1.8	−2.9±1.9
+dp/dt (mmHg/s)	SF	−53.4±51.8	−86.4±45.9	−115.1±74
	RG	−25.2±108.0	−128.0±87.3	−163.0±139.2
	RA	−21.4±30.4	−53.5±18.2	−58.1±26.6
−dp/dt (mmHg/s)	SF	54.6±34	71.1±42.6	107.5±62.7
	RG	25.3±62.6	149.0±104.5	183.4±131.4
	RA	32.2±26.9	51.9±21.6	51.7±22.1
				
Precontracted With U46619				
HR (beats/min)	SF	−1.3±7.1	−4.3±9.3	−3.5±5.2
	RG	−4.4±5.5	−2.5±18.9	3.6±19.7
	RA	−3.3±1.7	−6.0±3.3	−4.9±4.1
LVDP (mmHg)	SF	−0.1±7.8	−2.9±4.6	0.0±2.9
	RG	0.2±8.5	−1.4±5.2	2.4±3.3
	RA	3.5±5.2	−1.1±3.0	1.6±2.3
+dp/dt (mmHg/s)	SF	−36.2±78.4	−67.9±67.5	−22.8±43.8
	RG	−13.4±89.5	−43.9±69.9	9.9±50.7
	RA	25.4±54.5	−14.6±31.4	−5.7±30.5
−dp/dt (mmHg/s)	SF	43.6±58.0	43.7±50.7	21.5±36.1
	RG	16.9±62.9	29.1±46.0	8.1±25.8
	RA	−24.8±49.2	27.7±22.2	19.8±21.4

When the coronary vasculature was pre-contracted with U46619, this concentration-dependent decrease of CPP was more obvious than at the basal coronary resting tone. The effect of SF, RG and RA show no significant difference in CPP adjustment (figure 2B). SF/RG/RA injection caused small, non-signifcant increases or decreases in heart rate, LVDP, +dp/dt_max_ and −dp/dt_max_ when the coronary vasculature pre-contracted with U46619 (table 1).

### 3.3 The underlying mechanisms of coronary effects of SF, RG and RA

**Figure 3:**
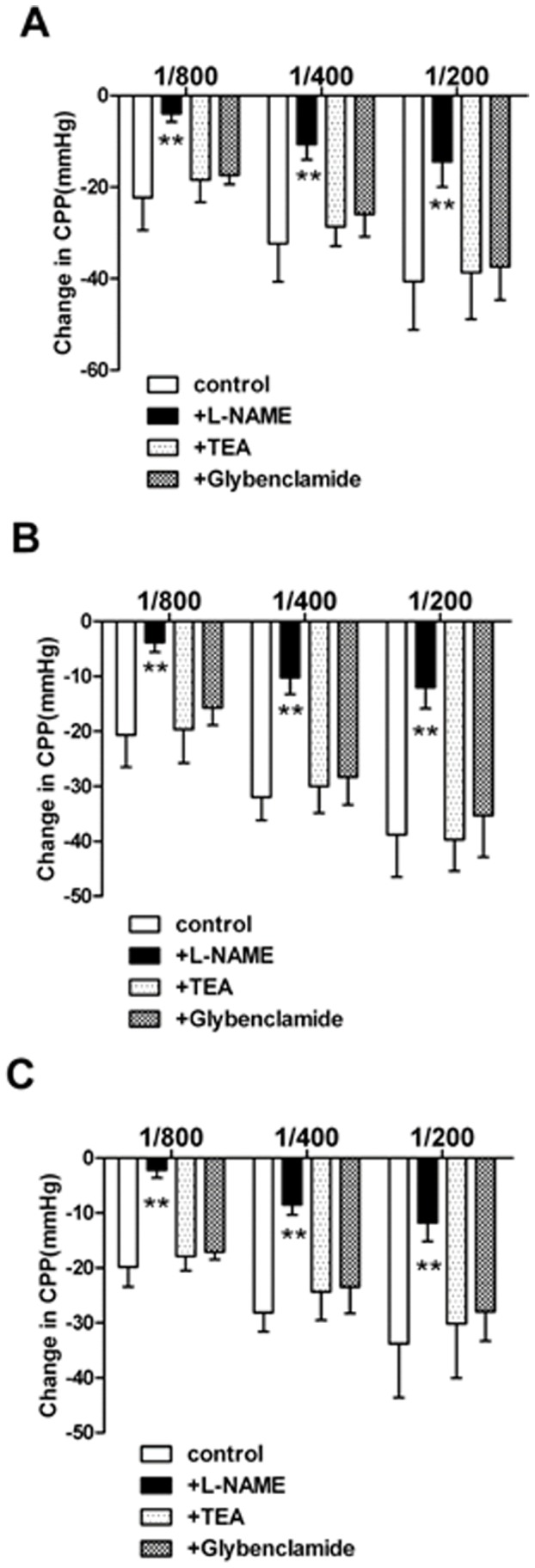
CPP change in perfused guinea pig hearts precontracted with U46619, after administration of SF (figure 3A), RG (figure 3B) and RA (figure 3C) in the presence or the absence (control, n = 6) of L-NAME(10^−4^ M, n = 6), TEA(10^−3^ M, n = 6), or glybenclamide(10^−5^ M, n = 6). Compared with other antagonist TEA and Glybenclamide, L-NAME can blunt the effect of CPP decrease driven by SF, RG and RA injections. Three titrations had been tested in each injection (1/800, 1/400, 1/200 dilution from commercial available injections). Values represent the mean ± SD. ** *P*<0.01 versus control.

In the hearts treated with L-NAME(10^−4^ mol/L), the NOS inhibitor, and precontracted with U46619, the strong coronary vasodilator response to SF(figure 3A), RG(figure 3B), and RA(figure 3C) injection were mostly abolished.

The coronary response to three kinds of injection were recorded after precontracting coronary vasculature with U46619, in the presence the blocker of Ca^2+^-dependent potassium channels, tetraethylammonium (TEA, 10^−3^ mol/L). SF(figure 3A), RG(figure 3B) and RA(figure 3C) reduced CPP in a manner similar to that observed in U46619-treated hearts(control).

In the presence the blocker of adenosine triphosphate-sensitive potassium channels (glybenclamide 10^−5^ mol/L), the relaxation to SF (figure 3A), RG(figure 3B) and RA(figure 3C) injection in hearts precontracted with U46619 was not roughly modified compared with untreated hearts (control).

### 3.4 SF injection and its two main components up-regulate eNOS expression

**Figure 4:**
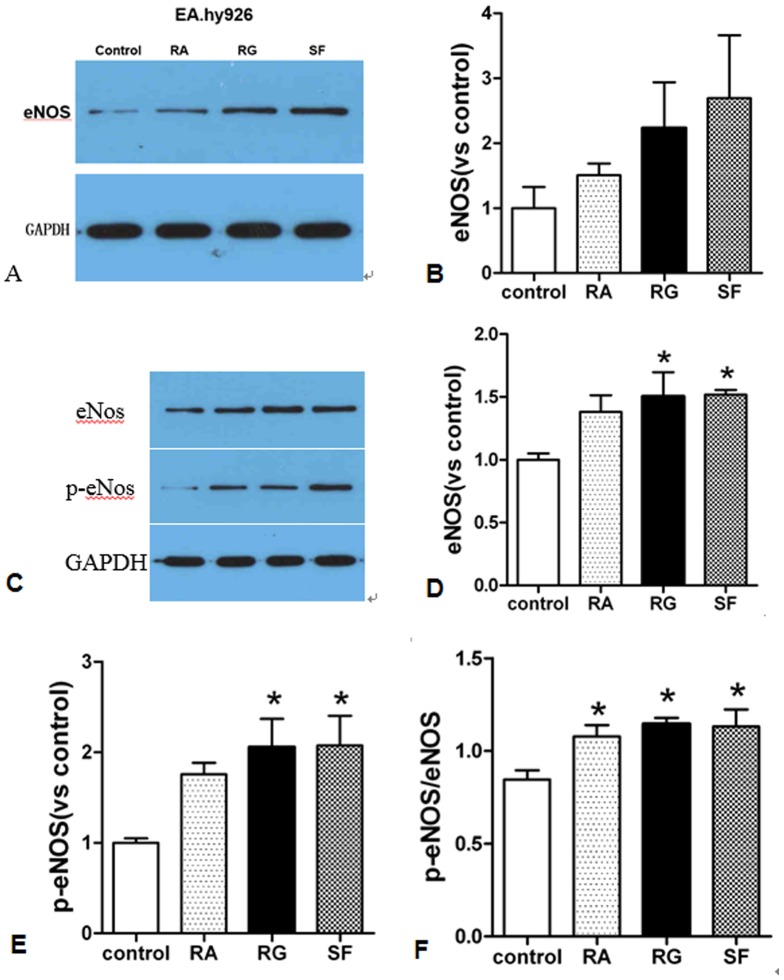
SF and its two main components RA and RG upregulate eNOS expression in EA.hy926 cells as show in immmunoblot (4A). 4B is the stastistic bar derived from grey brightness level of three independent immunoblots. 4C shows SF, RA and RG up-regulate eNOS and p-eNOS in rat cardiac microvascular endothelium cells. 4D and 4E is the statistic bar derived from grey brightness level of 4C (N = 3). 4F shows p-eNOS was increased more than total eNOS with SF, RA and RG treatment with statistical significance (P<0.05). Values represent the mean ± SEM.

Endothelial NOS is a nitric oxide synthase that generates NO in blood vessels and is involved in regulating vascular tone by inhibiting smooth muscle contraction [44]. Here we measuring expression of eNOS to assess alternation of NO level by SF, RG, RA administration. As shown in Figure 4, by contrasting to cells with no administration of medication (Control), 24 hours administration of SF injection (1/200) upregulated eNOS expression in EA.hy926 cells and in rat cardiac microvascular endothelium cells (RCMEC). Consistent with the report that production of nitric oxide depend on phosphorylation of eNOS in endothelium cells [47], we found that phosphated eNOS was increased more prominnent than total eNOS upon SF, RA and RG treatment. Comparing its two main components (both 24 hours administration and 1/200 dilution), RG presented stronger upregulation effect than RA. SF shows accumulation effect of RA with RG in eNOS regulation.

## Discussion

In this paper, we employed isolated Langendorff heart model which is well known for studying pharmacological interventions on myocardial function, vascular reactivity, electrical conduction and is able to be perfused using constant pressure or constant flow [25]. Coronary perfusion pressure, which indicates coronary resistance, is a sensitive parameter that is easily monitored. So when the isolated hearts were perfused with constant flow, CPP decreases when the coronary artery dilates [24]. Therefore constant flow perfusion is particularly well suited for studying the effect of vasoactive substances on coronary vasomotor tone [26]–[28]. The heart maintains the ability to autoregulate coronary vascular tone in constant pressure mode. Autoregulatory mechanisms strive to increase coronary flow under increased workload conditions. So it is applicable to study the positive inotropic drug [29]–[31].

Our present results suggest that Shenfu injection produced coronary vasodilatation in isolated perfused hearts reflected by increasing flow in constant pressure mode and decreasing coronary resistance in constant flow mode. The data supports that with constant pressure, SF injection can improve the contractility of the heart indicated by rise of LVDP and RPP. This is worked probably through dilating coronary artery with more supplied blood therefore more oxygen to the heart. This phenomenon is called “Gregg effect” [32] or the “garden-hose effect” [33] in which stimulated increases in coronary microvascular filling or perfusion pressure are associated with an enhancement of ventricular contractility. Without significant effect on the HR by SF injection suggests additional benefit of no further increase in myocardial oxygen demands. This is in accordance with the study using dog *in vivo* that SF injection improved the myocardium contractility and augmented cardiac output without raising the oxygen consume [34]. In summary, results of the present findings provide interesting information indicating that SF injection exerts a protective action with positive inotropy on isolated heart via improved CF. On the contrary, SF injection decrease LVDP and RPP followed by drop of CPP with constant flow suggesting reducing oxygen consumption. These changes shows that SF injection may be not exert direct effects on the ventricular muscle itself. Our results demonstrated that SF injection and its two components RG injection, RA injection respectively induced coronary vasodilatation in perfused hearts when the coronary vasculature at basal resting tone or precontracted with U46619. U46619, as a thromboxane A_2_ (TP) receptor agonist, can increase coronary perfusion pressure in constant flow mode through increasing phosphoinositide metabolism provoked by stimulating thromboxane A2 receptors [35]. It was reported that TP receptor activation can leads to generation of O_2_^−^ which is responsible for vasoconstriction. When the coronary vessel was precontracted by U46619, three injections produced more significant coronary vasodilatation than at basal tone in different physiological concentrations (1/800, 1/400 and 1/200). This modified vascular relaxation in the perfused heart attributes to the changes of artery tone [27]. However compared with SF injection and RG injection, RA injection did not demonstrate as strong as vasodilator effect unless hearts were pretreated by U46619. This disparity could be explained by the report that Fu Pian Injection whose components is RA had no significant inhibitory effect on KCl-induced contraction of thoracic aorta ring with intact endothelium as SF injection and RG injection [43]. So RA does not show apparent vasodilator role as SF and RG do. The myocardial contractility decreased accordingly resulted from significant reduced CPP at basal resting tone, measured as changes in LVDP and ±dp/dt. However no consistent significant HR responses were observed. The reduced myocardial contractility responses was abolished when the coronary artery was precontracted by U46619, which probably because U46619 exerts a positive inotropic effect on the heart resulted from the increase in phosphoinositide metabolism [36]–[37]. What's more, only L–NAME but not the Ca^2+^-activated potassium channel blocker (tetraethylammonium, TEA) nor the adenosine triphosphate (ATP)-sensitive potassium channel blocker (glybenclamide) can attenuate the vasodilator effects of SF injection, RG injection and RA injection, being pretreated with U46619. With the same concentration, L–NAME blunt vasodilation effect in an inversely dose dependent manner to subsequently added injections. Therefore, we deduced that NO but not potassium channel blocker contributes to the vasodilation effects of SF injection and its two components. NO has been reported to mediate the relaxation response to ginsenosides, which is the effective ingredient in SF injection and RG injection, in human aortic endothelial cells [38], in porcine coronary arteries [39] or in isolated rat hearts [40]. Indeed, we detected an up-regulation of eNOS expression upon SF, RG or RA administration as shown in Figure 4. Our results of RA injection as a vasodilator of vessels via NO is in agreement with the others who observed a relaxing effect on rat aorta from aconitine [41]. Niu et al. found that the relaxing effect of aconite decoction on aorta is related to NO released [42].

In summary, SF injection at physiological concentration produced significant coronary vasodilatation, mediated by RG injection and RA injection via releasing NO. SF injection may be not exert direct effects on the ventricular muscle itself. The protective action with positive inotropy on isolated heart is related to improved coronary flow.

## References

[1] Luo XY, Zhang FR, He RM (2009) Efficacy of shenfu injection as adjuvant therapy in treating patients of ischemic cardiomyopathy with heart insufficiency. Zhongguo Zhong Xi Yi Jie He Za Zhi. 29(8): 685–7. 19848196

[2] Zheng CD, Min S, Chin J (2008) Cardioprotection of Shenfu Injection against myocardial ischemia/reperfusion injury in open heart surgery. Integr Med. 14(1): 10–6. 10.1007/s11655-008-0010-y 18568324

[3] Wen-Ting S, Fa-Feng C, Li X, Cheng-Ren L, Jian-Xun L (2012) Chinese medicine shenfu injection for heart failure: a systematic review and meta-analysis. Evid Based Complement Alternat Med. 2012: 713149. 22611430 10.1155/2012/713149 PMC3348640

[4] Cao J, Zheng CD, Zhang GX, Zhang YJ, Min S (2005) Protective effect of Shenfu injection on myocardial mitochondria injured by ischemia-reperfusion in rabbits. Chin Med J (Engl). 118 (6): 505–7. 15788134

[5] KT Choi (2008) Botanical characteristics, pharmacological effects and medicinal components of Korean Panax ginseng C A Meyer. Acta Pharmacologica Sinica 29(9): 1109–1118. 18718180 10.1111/j.1745-7254.2008.00869.x

[6] Han JY, Ahn SY, Oh EH, Nam SY, Hong JT, et al (2012) Red ginseng extract attenuates kainate-induced excitotoxicity by antioxidative effects. Evid Based Complement Alternat Med. 2012: 479016. 23133495 10.1155/2012/479016 PMC3485976

[7] Bak MJ, Hong SG, Lee JW, Jeong WS (2012) Red ginseng marc oil inhibits iNOS and COX-2 via NFκB and p38 pathways in LPS-stimulated RAW 264.7 macrophages. Molecules. 17(12): 13769–86. 23174895 10.3390/molecules171213769 PMC6268309

[8] Chen IJ, Chang MY, Chiao SL, Chen JL, Yu CC, et al (2012) Korean red ginseng improves blood pressure stability in patients with intradialytic hypotension. Evid Based Complement Alternat Med. 2012: 595271. 22645630 10.1155/2012/595271 PMC3356894

[9] Li HX, Han SY, Ma X, Zhang K, Wang L, et al (2012) The saponin of red ginseng protects the cardiac myocytes against ischemic injury in vitro and in vivo. Phytomedicine. 19(6): 477–83. 22341690 10.1016/j.phymed.2012.01.002

[10] Shibata S, Fujita M, Itokawa H, Tanaka O, Ishii T (1963) Studies on the constituents of Japanese and Chinese crude drugs. Xi. Panaxadiol, a Sapogenin of Ginseng Roots. Chemical & Pharmaceutical Bulletin. 11: 762–765. 14068710 10.1248/cpb.11.759

[11] Yoshizaki K, Yahara S (2012) New triterpenoid saponins from fruits specimens of Panax japonicus collected in Kumamoto and Miyazaki prefectures (1). Chem Pharm Bull (Tokyo). 60(3): 354–62. 22382416 10.1248/cpb.60.354

[12] Popovich DG, Yeo CR, Zhang W (2011) Ginsenosides Derived from Asian (Panax ginseng), American Ginseng (Panax quinquefolius) and potential cytoactivity. International Journal of Biomedical and Pharmaceutical sciences. 56–62.

[13] Chen X (1996) Cardiovascular protection by ginsenosides and their nitric oxide releasing action. Clin Exp Pharmacol Physiol. 23(8): 728–32. 8886498 10.1111/j.1440-1681.1996.tb01767.x

[14] Deng J, Wang YW, Chen WM, Wu Q, Huang XN (2010) Role of nitric oxide in ginsenoside Rg(1)-induced protection against left ventricular hypertrophy produced by abdominal aorta coarctation in rats. Biol Pharm Bull 33(4): 631–5. 20410597 10.1248/bpb.33.631

[15] Peng L, Sun S, Xie LH, Wicks SM, Xie JT (2012) Ginsenoside Re: pharmacological effects on cardiovascular system. Cardiovasc Ther. 30(4): e183–8. 21884006 10.1111/j.1755-5922.2011.00271.x

[16] Zhao D, Wang J, Cui Y, Wu X (2012) Pharmacological effects of Chinese herb aconite (fuzi) on cardiovascular system. J Tradit Chin Med. 32(3): 308–13. 23297548 10.1016/s0254-6272(13)60030-8

[17] Ji XF, Yang L, Zhang MY, Li CS, Wang S, et al (2011) Shen-Fu injection attenuates postresuscitation myocardial dysfunction in a porcine model of cardiac arrest. Shock. 35(5): 530–536. 21263380 10.1097/SHK.0b013e31820e2058

[18] Zhou SJ, Du GY (2003) Effects of higenamine on the cardio-circulatory system. Zhongguo Zhong Yao Za Zhi. 28(10): 910–3. 15620176

[19] Morrison RR, Teng B, Oldenburg PJ, Katwa LC, Schnermann JB, et al (2006) Effects of targeted deletion of A1 adenosine receptors on postischemic cardiac function and expression of adenosine receptor subtypes. Am J Physiol Heart Circ Physiol. 291(4): H1875–H1882. 16679400 10.1152/ajpheart.00158.2005

[20] Skrzypiec-Spring M, Grotthus B, Szelag A, Schulz R (2007) Isolated heart perfusion according to Langendorff-Still viable in the new millennium. Journal of Pharmacological and Toxicological Methods 55, 113–126. 10.1016/j.vascn.2006.05.006 16844390

[21] Meng X, Ao L, Brown JM, Fullerton DA, Banerjee A, et al. (1997) Nitric oxide synthase is not involved in cardiac contractile dysfunction in a rat model of endotoxemia without shock. Shock. 7: 111–118. 10.1097/00024382-199702000-00007 9035287

[22] Ponto LL, O'Leary DS, Koeppel J, Block RI, Watkins GL, et al. (2004) Effect of acute marijuana on cardiovascular function and central nervous system pharmacokinetics of [^15^O] water: effect in occasional and chronic users. Journal of Clinical Pharmacology. 44: 751–766. 10.1177/0091270004265699 15199080

[23] Stowe DF, Graf BM, Fujita S, Gross GJ (1996) One-day cold perfusion of bimakalim and butanedione monoxime restores ex situ cardiac function. American Journal of Physiology. 271: 1884–1892. 10.1152/ajpheart.1996.271.5.H1884 8945905

[24] McLean PG, Aston D, Sarkar D, Ahluwalia A (2002) Protease-activated receptor-2 activation causes EDHF-like coronary vasodilation: selective preservation in ischemia/reperfusion injury: involvement of lipoxygenase products, VR1 receptors, and C-fibers. Circulation Research. 90: 465–472. 10.1161/hh0402.105372 11884377

[25] Guo L, Dong Z, Guthrie H (2009) Validation of a guinea pig Langendorff heart model for assessing potential cardiovascular liability of drug candidates. J Pharmacol Toxicol Methods. 60(2): 130–51. 19616638 10.1016/j.vascn.2009.07.002

[26] García-Villalón AL, Fernández N, Monge L, Diéguez G (2011) Coronary response to diadenosine tetraphosphate after ischemia-reperfusion in the isolated rat heart. Eur J Pharmacol. 660(2–3): 394–401. 21513710 10.1016/j.ejphar.2011.04.006

[27] Montoya JJ, Fernández N, Monge L, Diéguez G, Villalón AL (2011) Nitric oxide-mediated relaxation to lactate of coronary circulation in the isolated perfused rat heart. J Cardiovasc Pharmacol. 58(4): 392–8. 21697724 10.1097/FJC.0b013e318226bcf7

[28] Favaloro JL, Kemp-Harper BK (2007) The nitroxyl anion (HNO) is a potent dilator of rat coronary vasculature. Cardiovasc Res. 73(3): 587–96. 17189622 10.1016/j.cardiores.2006.11.018

[29] Liao R, Podesser BK, Lim CC (2012) The continuing evolution of the Langendorff and ejecting murine heart: new advances in cardiac phenotyping. Am J Physiol Heart Circ Physiol. 303(2): H156–H167. 22636675 10.1152/ajpheart.00333.2012 PMC3404701

[30] Mishra RC, Belke D, Wulff H, Braun AP (2013) SKA-31, a novel activator of SK(Ca) and IK(Ca) channels, increases coronary flow in male and female rat hearts. Cardiovasc Res. 97(2): 339–48. 23118129 10.1093/cvr/cvs326 PMC3543990

[31] Liu CH, Chen MF, Tseng TL, Chen LG, Kuo JS, et al (2012) Oroxylin A, but Not Vasopressin, Ameliorates Cardiac Dysfunction of Endotoxemic Rats. Evid Based Complement Alternat Med. 2012: 408187. 23193421 10.1155/2012/408187 PMC3489109

[32] Gregg DE (1963) Effect of coronary perfusion pressure or coronary flow on oxygen usage of the myocardium. Circulation Research. 13: 497–500. 14120967 10.1161/01.res.13.6.497

[33] Arnold G, Kosche F, Miessner E, Neitzert A, Lochner W (1968) The importance of the perfusion pressure in the coronary arteries for the contractility and the oxygen consumption of the heart. Pflugers Arch Gesamte Physiol Menschen Tiere 299(4): 339–56. 10.1007/BF00602910 5247223

[34] Yang FJ, Wang ZR, Lin DP, Qu Y, Yin HH, et al (2003) The influence on hemodynamics of myocardial ischemic dogs and blood pressure of animals with shenfu injection. Zhongguo Zhong Yao Za Zhi. 28(3): 259–62. 15015316

[35] Sakuma I, Gross SS, Levi R (1989) Positive inotropic effect of the thromboxane analog U-46619 on guinea pig left atrium: mediation by specific receptors and association with increased phosphoinositide turnover. Can J Physiol Pharmacol. 67(8): 943–949. 2557146 10.1139/y89-148

[36] Mantelli L, Amerini S, Rubino A, Ledda F (1992) Effects of thromboxane agonists on cardiac adrenergic neurotransmission. Eur J Pharmacol. 213(1): 79–85. 1323473 10.1016/0014-2999(92)90235-v

[37] Dubé GP, Jakubowski JA, Brune KA, Bemis KG, Kurtz WL (1995) *In vivo* effects of a novel thromboxane A2/prostaglandin H2 (TXA2/PGH2) partial agonist (+)5(Z)-7-[3-endo-phenylsulfonylamino[2.2.1]-bicyclohept-2-exo-yl]-heptenoic acid [(+)-S-145], on vascular, platelet and cardiac function. J Pharmacol Exp Ther. 272(2): 799–807. 7853197

[38] Yu J, Eto M, Akishita M, Kaneko A, Ouchi Y, et al (2007) Signaling pathway of nitric oxide production induced by ginsenoside Rb1 in human aortic endothelial cells: a possible involvement of androgen receptor. Biochem Biophys Res Commun. 353(3): 764–9. 17196933 10.1016/j.bbrc.2006.12.119

[39] Zhou W, Chai H, Lin PH, Lumsden AB, Yao Q, et al (2005) Ginsenoside Rb1 blocks homocysteine-induced endothelial dysfunction in porcine coronary arteries. J Vasc Surg. 41(5): 861–8. 15886672 10.1016/j.jvs.2005.01.054

[40] Yi XQ, Li T, Wang JR, Wong VK, Luo P, et al (2010) Total ginsenosides increase coronary perfusion flow in isolated rat hearts through activation of PI3K/Akt-eNOS signaling. Phytomedicine. 17(13): 1006–15. 20724124 10.1016/j.phymed.2010.06.012

[41] Chong WS, Lee YS, Kang YJ (1998) Comparison of Inodilator Effect of Higemanine, YS-49, YS-51,Tetrahydroisoquinoline Analogs, and Dobutamine in the Rat. Korean J Physiol Pharmacol 2(3): 323–330.

[42] Niu CQ, Zhang TX, Xu HQ, Qin XM (2004) Research of vasodilation effect of aconite decoction on the rabbit aorta *in vitro* . Zhong Yao Yao Li Yu Lin Chuang 20(4): 23–25.

[43] Zhu J, Kang L, Ye Q, Fan G, Liang Y, et al. (2013) Effects of shenfu injection and its main components on the contraction of isolated rat thoracic aortic rings. PLoS One. 30; 8 (10) 10.1371/journal.pone.0078026 PMC3813522 24205074

[44] Yoshimura M, Yasue H, Nakayama M, Shimasaki Y, Sumida H, et al (1998) A missense Glu298Asp variant in the endothelial nitric oxide synthase gene is associated with coronary spasm in the Japanese. Hum Genet. 103(1): 65–9. 9737779 10.1007/s004390050785

[45] Shouyuan T, Zhijia G, Jiangang W, Jie ZW, Fang HC, et al (2009) The Protective Effects of Pretreatment with Shenfu Injection on Myocardial Ischemia/reperfusion Injury in Dogs. Chinese Journal of Integrative Medicine on Cardio-/Cerebrovascular Disease. 7 (11): 1317–1319.

[46] ZeLong H, WeiHong Y, XiaoLing Z (2001) Effect and mechanism of Shen Fu Injection on hemodynamics of congestive heart failure patient. Chinese Journal of Integrative Medicine. 21 (5): 386.

[47] Hambrecht R, Adams V, Erbs S, Linke A, Kränkel N, et al (2003) Regular physical activity improves endothelial function in patients with coronary artery disease by increasing phosphorylation of endothelial nitric oxide synthase. Circulation. 107(25): 3152–8. 12810615 10.1161/01.CIR.0000074229.93804.5C

[48] Nishida M, Carley WW, Gerritsen ME, Ellingsen O, Kelly RA, et al (1993) Isolation and characterization of human and rat cardiac microvascular endothelial cells. Am J Physiol. 264(2 Pt 2): H639–52. 8447476 10.1152/ajpheart.1993.264.2.H639

